# Linking Mechanisms of Vitamin D Signaling with Multiple Sclerosis

**DOI:** 10.3390/cells12192391

**Published:** 2023-09-30

**Authors:** Carsten Carlberg, Marcin P. Mycko

**Affiliations:** 1 Institute of Animal Reproduction and Food Research, Polish Academy of Sciences, 10-748 Olsztyn, Poland; 2 Institute of Biomedicine, School of Medicine, University of Eastern Finland, 70211 Kuopio, Finland; 3 Department of Neurology, Laboratory of Neuroimmunology, University of Warmia and Mazury in Olsztyn, Warszawska 30, 10-082 Olsztyn, Poland; marcin.mycko@uwm.edu.pl

**Keywords:** multiple sclerosis, vitamin D, vitamin D response index, immune system, genetics, epigenetics

## Abstract

Environmental triggers often work via signal transduction cascades that modulate the epigenome and transcriptome of cell types involved in the disease process. Multiple sclerosis (MS) is an autoimmune disease affecting the central nervous system being characterized by a combination of recurring inflammation, demyelination and progressive loss of axons. The mechanisms of MS onset are not fully understood and genetic variants may explain only some 20% of the disease susceptibility. From the environmental factors being involved in disease development low vitamin D levels have been shown to significantly contribute to MS susceptibility. The pro-hormone vitamin D_3_ acts via its metabolite 1α,25-dihydroxyvitamin D_3_ (1,25(OH)_2_D_3_) as a high affinity ligand to the transcription factor VDR (vitamin D receptor) and is a potent modulator of the epigenome at thousands of genomic regions and the transcriptome of hundreds of genes. A major target tissue of the effects of 1,25(OH)_2_D_3_ and VDR are cells of innate and adaptive immunity, such as monocytes, dendritic cells as well as B and T cells. Vitamin D induces immunological tolerance in T cells and reduces inflammatory reactions of various types of immune cells, all of which are implicated in MS pathogenesis. The immunomodulatory effects of 1,25(OH)_2_D_3_ contribute to the prevention of MS. However, the strength of the responses to vitamin D_3_ supplementation is highly variegated between individuals. This review will relate mechanisms of individual’s vitamin D responsiveness to MS susceptibility and discuss the prospect of vitamin D_3_ supplementation as a way to extinguish the autoimmunity in MS.

## 1. Introduction

The central nervous system (CNS) autoimmune disease MS is the leading cause of disability in younger and middle-aged adults that is not caused by a trauma [1]. The clinically most common form of the disease is RRMS (relapsing remitting MS), i.e., an alternation between symptomatic attacks (relapses) and periods of recovery (remissions), which is found in up to 90% of patients in the age range of 20–40 years [2]. When RRMS progresses, the patient accumulates irreversible neurological disabilities. The main pathological feature of MS are focal lesions in the CNS, which are created by B and T cells from the periphery that pass a disrupted blood-brain barrier and act against an antigen in the CNS [3]. The best candidate for the latter is myelin, which is a protein that insulates and protects axons, so that they can efficiently conduct nerve impulses. The loss of myelin as well as the prevention of remyelination due to increasingly disabled myelin-producing oligodendrocytes leads to axon destruction and neurodegeneration. This process is associated with waves of inflammation, i.e., the massive production of pro-inflammatory cytokines. The latter is triggered by CD4^+^ cells that are polarized as T helper (T_H_) cells of types 1 and 17 [4]. Thus, MS is an inflammatory autoimmune disease that is based on the interaction of (i) T and B cells of adaptive immunity, (ii) dendritic cells, monocytes, natural killer cells and microglia of innate immunity and (iii) oligodendrocytes, astrocytes and neurons of the CNS. Although several successful clinical trials have been completed in MS demonstrating that immunosuppressing and immunomodulatory therapies can lead to slowing down this disease, a fully effective treatment is still lacking. Accordingly, MS is currently still an incurable disease and even newest therapies may fail in stopping disease progression or lead to the severe complications [5]. Therefore, maximal attention should be taken on preventing disease onset.

**Figure 1:**
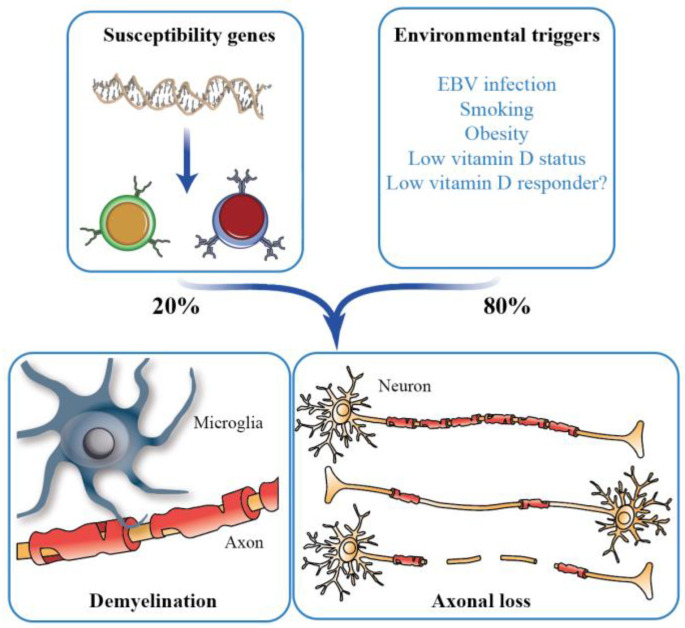
MS and vitamin D. MS is a complex autoimmune disorder, the onset of which is triggered by a combination of genetic (with estimated contribution 20%) and environmental factors (with estimated contribution 80%). MS is characterized by inflammatory, demyelinating lesions in the CNS leading to heterogeneous axonal loss.

In general, the susceptibility for most common, non-communicable diseases like MS is based on a complex interplay of genetics and environment [6]. This means that both inherited or acquired genetic variants as well as environmental signals that via signal transduction pathways affect the function of transcription factors and chromatin modifiers [7] contribute to the disease risk. SNVs (single nucleotide variants) associated with MS susceptibility highlight genes in their vicinity that have key roles in immunity [8]. The cluster of *HLA* (human leukocyte antigen) genes, which encode for type I and type II MHC (major histocompatibility complex) proteins, are a “hotspot” for MS-related genes. The most prominent genetic risk factor for MS is the HLA-DRB*15:01 gene variant [9]. The gene encodes for a MHC type II protein, which is expressed on antigen-presenting cells and binds endogenous myelin autoantigens and probably also MS-associated foreign antigens [10]. Although currently more than 200 identified MS-related SNVs have been described, together they explain only some 20% of the disease risk [8]. Therefore, environmental factors seem to play a major role in MS susceptibility, such as EBV (Epstein Barr virus) infection. In addition, the lifestyle of the individual, such as smoking (promoting via lung inflammation systemic activation of proinflammatory pathways), adolescent obesity (causing systemic low grade chronic inflammation) and the frequency of outdoor activities (increasing endogenous vitamin D_3_ production via sun exposure), increases or decreases the risk of MS onset [11,12,13] (Figure 1).

**Figure 2:**
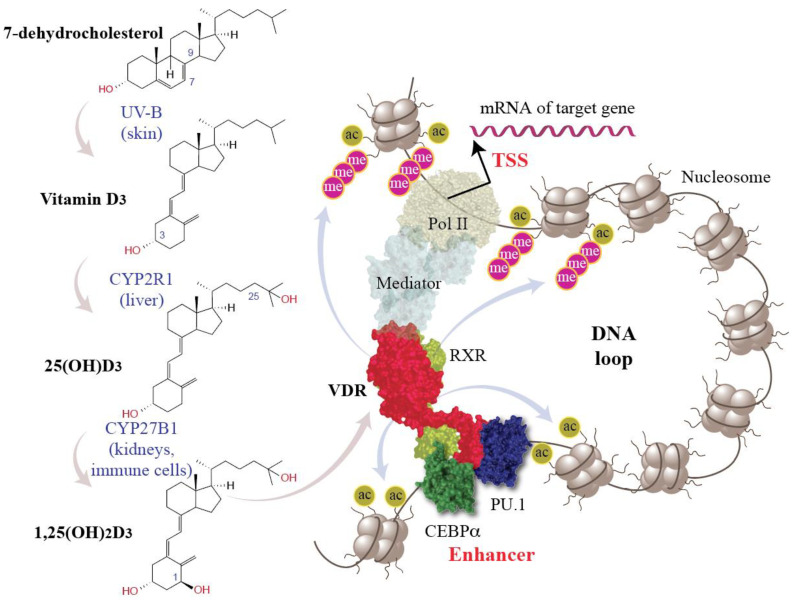
Principles of vitamin D signaling. 7-dehydrolesterol is a direct precursor of cholesterol but under exposure with UV-B can also convert into vitamin D_3_ (left). CYP2R1 (cytochrome P450 family 2 subfamily R member 1) hydroxylates vitamin D_3_ into 25(OH)D_3_ (25-hydroxyvitamin D_3_) and CYP27B1 adds a further hydroxy group to C1. 1,25(OH)_2_D_3_ is a nuclear hormone that activates VDR (red). The pioneer transcription factors PU.1 (purine-rich box 1, dark blue) and CEBPα (CCAAT enhancer binding protein α, green) support the DNA binding of VDR-RXR (retinoid X receptor, light green) heterodimers. VDR-bound enhancers activate via DNA looping and the Mediator complex Pol II (RNA polymerase II) waiting on the TSS (transcription start site) region of a vitamin D target gene. This finally changes the expression of hundreds of vitamin D target genes (top right), a number of which are involved in the regulation of the immune system and have an impact on MS (Section 4).

A low vitamin D status, which often relates to low sun exposure, is another important environmental risk for MS [2,9,14]. In contrast, sufficient vitamin D levels, in particular within the first decades of life, are protective against disease onset [15]. Vitamin D_3_ is a pro-hormone being produced endogenously in UV-B exposed human skin [16,17] (Figure 2). However, the seasonal variation of sun exposure affects in particular to those individuals living above a latitude of 38° N (15% of world population) [18,19]. In addition, in modern lifestyle most individuals spend long times indoors, while for climatic and cultural reasons they are covering most of our skin outdoors. Therefore, in particular in Europe, vitamin D_3_ needs to be supplemented during winter times, in order not to develop vitamin D deficiency.

Vitamin D is best known for its regulation of calcium homeostasis, which is important for proper bone mineralization [20,21], but in addition 1,25(OH)_2_D_3_ and VDR have a modulatory role on the immune system. Ligand-activated VDR stimulates innate immune cells like monocytes and macrophages, which fight against pathogenic microbes [22]. In parallel this inhibits overboarding responses of cells of the adaptive immune system [23,24], such as B and T cells, i.e., and prevents in this way the onset of autoimmune diseases like MS [25], rheumatoid arthritis [26], inflammatory bowel disease [27] and type I diabetes [28]. Importantly, the VITAL RCT (randomized controlled trial) with more than 25,000 participants indicated that vitamin D_3_ supplementation (2000 IU/day) over 5 years reduced the risk of autoimmune diseases by 22% [29]. This is an important finding, since for the primary target of the VITAL trial, the prevention of cancer and cardiovascular disease, there was a null result [30]. Furthermore, Mendelian randomization studies reported a significantly increased risk of a low vitamin D status for MS onset, while comparable studies focusing on other outcomes had null effects [31]. Thus, vitamin D seems to have more prominent effects on autoimmunity and in particular on MS than on many other diseases.

The ability of vitamin D to modulate the activity of immune system seems to be the key in understanding how its lack may cause a malfunction of immune cells in the periphery. Some of these incorrectly programmed immune cells then migrate to the CNS, where they target myelin. This short review describes current views on the role of vitamin D in MS, with a focus on its impact on the immune function and epigenetic landscape of the cells as a mechanism contributing to autoimmune demyelination.

## 2. Vitamin D Status versus Responsiveness

**Figure 3:**
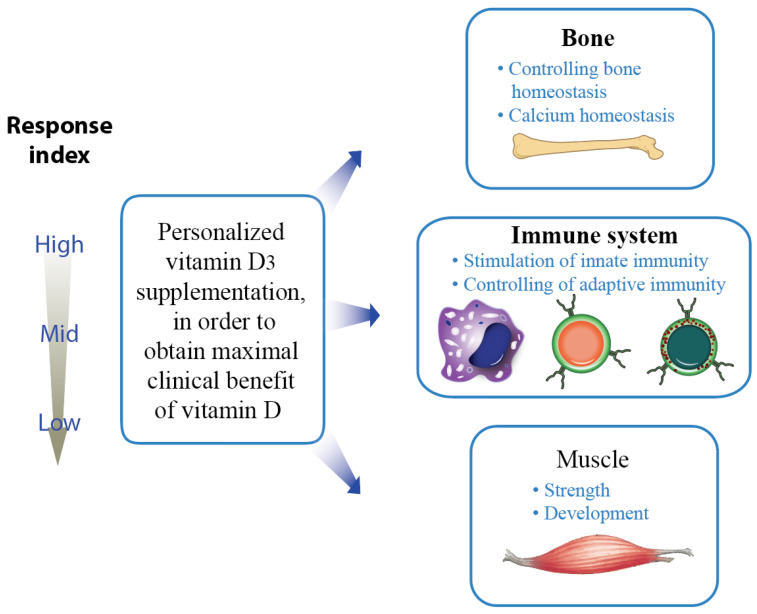
Knowing an individual’s vitamin D response index allows personalized supplementation with vitamin D_3_. In this way, clinical benefits of vitamin D are optimized.

The serum levels of the abundant vitamin D_3_ metabolite 25(OH)D_3_, which has a half-life of more than 14 days, are a biomarker for the vitamin D status [32]. Below a 25(OH)D_3_ level of 50 nM (20 ng/mL) an individual is considered as vitamin D deficient [33], because his/her risk for musculoskeletal disorders like rickets in children as well as osteomalacia and fractures in adults like is significantly increased [34]. Therefore, on the population level a vitamin D status in the range of 75–100 nM (30–40 ng/mL) 25(OH)D_3_ is recommended [35], in order to get musculoskeletal as well as non-skeletal benefits of vitamin D. For the average person, deficiency can be prevented by a supplementation of 25 µg (1000 IU) vitamin D_3_ per day [36], but a dose of 1 µg (40 IU)/kg body weight may be most appropriate, in order to fulfill also the needs of low vitamin D responders (Figure 3). In cases of severe deficiency, a bolus of 1000 IU vitamin D_3_/kg body weight can speed up reaching appropriate 25(OH)D_3_ levels. However, long-term overdosing of vitamin D_3_ or its metabolites should be avoided, in order not to cause tissue calcification through hypercalcemia.

Since there is significant genetic and epigenetic variation between human individuals [37], recommendations for vitamin D_3_ supplementation for the whole population may not be suited best for everyone. The concept of the vitamin D response index was developed on the basis of the vitamin D intervention studies VitDmet (NCT01479933, ClinicalTrials.gov (accessed on 14 June 2023)) [38,39,40,41] and VitDbol (NCT02063334) [42,43], which were designed more as a medical experiment than an RCT. In the VitDmet study, 71 elderly pre-diabetic individuals were supplemented daily with either 0, 40, or 80 µg vitamin D_3_ over 5 months during winter in Finland. Blood samples were collected at the beginning and end of the intervention. In contrast, the VitDbol study used 35 young healthy individuals, who obtained a single bolus of 80,000 IU vitamin D_3._ Samples were gathered at days 0, 1, 2, and 30. PBMCs (peripheral blood mononuclear cells) were isolated, and RNA was prepared without any further in vitro culture. The supplemented vitamin D_3_ is endogenously converted to 25(OH)D_3_ and 1,25(OH)_2_D_3_, which results in changes of gene expression being measured by qPCR (quantitative polymerase chain reaction) or RNA-seq (RNA sequencing). Thus, the interventions represent safe human in vivo experiments. 

**Figure 4:**
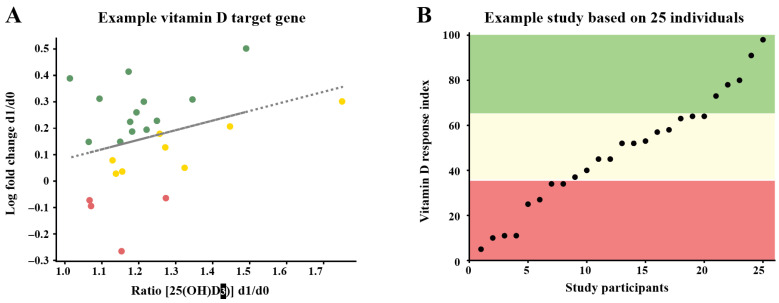
Principles of determining the vitamin D response index. Data are analyzed by relating the changes in the expression of vitamin D target genes at day 1 (d1) and day 0 (d0) to the ratio of the 25(OH)D_3_ serum levels at d1 and d0 (**A**). In this way, the response of the target genes is calculated comparably to in vitro studies [23]. The responsiveness of each the study participants is scored for every selected vitamin D target gene as no (red), weak (yellow) or strong responders (green) (**A**). The sums of the scores of all tested genes is ranked and the individuals are segregated by the method *k-means* into low, mid and high responders [44] (**B**).

Based on these studies, individuals can be distinguished via vitamin D-triggered parameters into high, mid and low responders to vitamin D (Figure 4). Importantly, 25% of the study participants showed a low vitamin D response index [44,45]. These individuals should take higher doses of vitamin D_3_ than suggested by guidelines for the general population (maximal 1000 IU, but in many countries far less). In contrast, persons with a high vitamin D response index are better prepared to cope with European winters characterized by low or no endogenous vitamin D_3_ production. This suggests that low vitamin D responders may suffer more frequently from autoimmune diseases [46], infections [47] and/or cancer [48], while high vitamin D responders have lower susceptibilities for these diseases. So far, there is no standardized procedure to determine the vitamin D response index. However, this may not be necessary, if all individuals are treated as potential low responders, i.e., supplemented with 4000 IU vitamin D_3_/day.

Like most other traits, the vitamin D response index is based on genetics, epigenetics and environment. Variants in the genes controlling vitamin D transport and metabolism, such as *DHCR7* (7-dehydrocholesterol reductase), *CYP2R1*, *CYP24A1* and *GC* (GC vitamin D binding protein), contribute to differences in the vitamin D status of individuals [49]. Interestingly, SNVs in the loci of the genes *CYP24A1*, *CYP2R1* and *DHCR7* contribute to MS susceptibility [50,51,52]. However, a person’s vitamin D response index is independent from his/her vitamin D status. Accordingly, high responders can have a low 25(OH)D_3_ serum levels and handle it well, while low responders should have a high vitamin D status, since at low 25(OH)D_3_ levels they cannot benefit from the physiological actions of vitamin D (Figure 3). In fact, high responders can handle a low vitamin D status, while low responders need to have a high vitamin D status, in order to benefit from vitamin D. Thus, genetic variants contributing to the vitamin D status and to the vitamin D response index are expected to be largely different.

On the molecular level the vitamin D response index is not well understood. In the case of the anti-coagulant drug warfarin interindividual differences in the response to its administration are found to be based on SNVs in the genes *CYP2C9* and *VKORC1* (vitamin K epoxide reductase complex subunit 1) [53]. In contrast, so far no SNVs related to the vitamin D response index have been described. This makes it likely that epigenetics and environment rather than genetics can explain this trait. It had been observed that persons differ in their sets of vitamin D target genes, which may be part of the explanation of differences in the vitamin D response index [54]. Moreover, vitamin D target genes have different EC_50_-value for their activation by vitamin D [55]. While some genes respond already at 0.1 nM 1,25(OH)_2_D_3_, others require levels of 1 nM and higher. The difference in the response of vitamin D target genes relates to their epigenetic status suggesting that also interindividual differences in the vitamin D response index are based on epigenetics.

## 3. Vitamin D Regulates the Epigenome and Transcriptome of Immune Cells

Chromatin is a complex of genomic DNA with nuclear proteins, such as histones, and serves as the physical expression of the epigenome. The epigenetic status of a gene, i.e., primarily the accessibility of its TSS and enhancer regions, determines, whether it can be transcribed or not. Thus, the epigenome regulates the transcriptome [56,57]. Interestingly, a number of attributes of the epigenome, such as (i) the accessibility of chromatin, (ii) the binding of transcription factors like VDR and the pioneer factors PU.1 and CEBPα, (iii) the binding of chromatin organizing factors, such as CTCF (CCCTC-binding factor), as well as changes in histone modifications are vitamin D sensitive [58,59] (Figure 2). There are some 10,000 VDR binding loci per *VDR* gene expressing cell type [60], i.e., there are thousands of sites within the epigenome where vitamin D may have an effect. However, only a minority of VDR-bound genomic regions contact via DNA looping TSS regions, i.e., they have an effect on the transcriptome [61]. Thus, the vast majority of genomic VDR binding sites seem to contribute to epigenetic memory of a cell but not directly to gene transcription.

**Figure 5:**
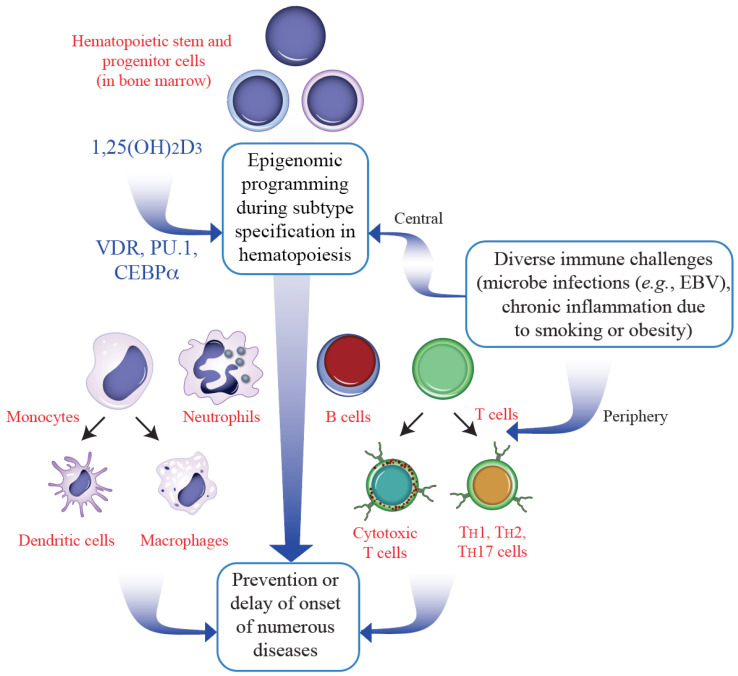
Hematopoietic differentiation triggered by vitamin D. The epigenome modulating effect of vitamin D (*via* the VDR with the help of PU.1 and CEBPα) modulates the differentiation of immune cells during hematopoiesis (**top**). In parallel, many immune challenges influence this differentiation process in the bone marrow as well as in the periphery. The stabilization of the epigenomes of the subtypes of monocytes and T cells (**bottom**) contributes to the prevention or delaying the onset of diseases including MS.

Hematopoiesis generates out of stem cell in the bone marrow more than 100 different cell types of the immune system and the blood. This involves epigenetic programming of terminally differentiated cells and creates a dominant form of epigenetic memory. Together with PU.1 and CEBPα VDR acts as a key regulator of myeloid line of hematopoiesis generating innate cells like granulocytes and monocytes [62] (Figure 5). Since most immune cell types have a rapid turnover, they can respond more flexibly to environmental changes than other tissues. Perturbations like antigen encounter and DAMPs (danger associated molecular patterns) exposure induce signal transduction cascades, the end of which are chromatin modifiers and transcription factors. This leads to changes in the epigenome and transcriptome of the respective immune cells representing another form of epigenetic memory known as trained immunity [63,64]. Thus, immune challenges are linked to epigenetic memory preparing the cells to react more effectively to a repeated encounter [65]. This may explain epidemiological observations that supplementation with vitamin D_3_ [66] or cod liver oil [67] at young age significantly reduces MS susceptibility later in life. Since the immune system takes about the first 10 years of life to mature, vitamin D sufficiency ensuring proper immune cell programming reduces the risk of MS onset (Figure 5).

## 4. Vitamin D Target Genes with Impact for MS

The cells of the immune system that are most responsive to vitamin D are monocytes [44], which via their differentiated forms, dendritic cells and macrophages, do not only modulate inflammation, but also metabolic pathways. The evolutionary oldest function of VDR is the regulation of genes involved of energy metabolism [68,69], such as *FBP1* (fructose-bisphosphatase 1) [70] and *PFKFB4* (6-phosphofructo-2-kinase/fructose-2,6-biphosphatase 4) [71]. The innate as well as the adaptive immunity both use a lot of energy for growth and differentiation, VDR became also an important regulator of immunity [72]. Thus, vitamin D connects (energy) metabolism with immunity [73,74].

Ligand-activated VDR inhibits the maturation, differentiation and stimulatory capacity of dendritic cells [75]. This involves reprogramming of glucose metabolism via upregulating the *PFKFB4* gene and changing the functional profile of dendritic cells. These epigenetic changes result in the reduction of T_H_1 cell counts and the increase of T_reg_ (T regulatory) and T_H_2 cells [25,76], which are critical for immunological tolerance. Moreover, during the development of autoimmune diseases like MS, the T_H_17/T_reg_ ratio gets into misbalance [77]. Vitamin D can (i) inhibit the polarization of T cells into the subtype T_H_17 by downregulating the genes *IL17* (interleukin 17), *IL22*, *RORC* (RAR related orphan receptor C) and *IL23R* (interleukin 23 receptor) [78] and (ii) support the production of T_reg_ cells by upregulating the genes *FOXP*3 (forkhead box P3), *IL10* and *CTLA4* (cytotoxic T-lymphocyte associated protein 4) [79]. In early stages of MS, T_H_1 cell numbers are high and mediate chronic inflammation causing demyelination [80,81]. In contrast, ligand-activated VDR antagonizes the pro-inflammatory transcription factors NFκB (nuclear receptor κB) and NFAT (nuclear receptor activated T cells) so that the *IL2* gene, which is the major growth factor adaptive immunity, is produced in lower amounts [82].

Short-term vitamin D_3_ bolus supplementations can be used as safe human in vivo experiments. In this context of such experiments, at some 800 genomic regions significant changes in chromatin accessibility were observed [83]. Interestingly, the *HLA* gene cluster, shows a high density of vitamin D-triggered chromatin accessibility changes. 

Furthermore, 10 of the 12 genes within the *HLA* class II subcluster were classified as vitamin D targets in PBMCs [23]. In addition, in monocytes also the class I genes *HLA-A* and *HLA-C* respond to vitamin D [84]. Thus, not only SNVs like HLA-DRB*15:01 [9] highlight the link between the *HLA* gene cluster and MS susceptibility, but vitamin D-triggered changes in the epigenome and transcriptome are also important.

Since MS is a complex multigenic disease, there are many other vitamin D target genes related to the disease. For example, the effects of vitamin D on the growth of hematopoietic stem cells is mediated by the family of *CXCL* (C-X-C motif chemokine ligand) genes [85], of which *CXCL1*, *CXCL5*, *CXCL7*, *CXCL8*, *CXCL9*, *CXCL10*, *PARM1* (prostate androgen-regulated mucin-like protein 1) and *EREG* (epiregulin) are vitamin D targets [23]. Other vitamin D target genes related to autoimmunity are *ACVRL1* (activin A receptor like type 1), *NINJ1* (ninjurin 1), *SRGN* (serglycin), *CD93* (CD93 molecule) and *CEBPB* [86]. Interestingly, the latter genes are highly expressed in immune cells but show only low inducibility. The long-term response of these genes corresponds with the low vitamin D responsiveness of their enhancers [86]. These observations suggest that vitamin D target genes involved in autoimmunity have a different set of vitamin D-triggered enhancers than those that contributing to rapid responses to microbe infections [87]. All these data strongly implicate a causal link between vitamin D induced transcriptional changes and the development of MS.

## 5. Relating MS Pathogenesis to Vitamin D

The pathology of MS can be subdivided into the processes (i) activation of self-reactive B and T cells, (ii) invasion of the CNS by these immune cells through the disruption of the blood-brain barrier, (iii) effector function of the invaded cells and (iv) progressive neurodegeneration [2]. The main role of vitamin D is to prevent the onset of MS through a lifelong support of proper epigenetic programing of immune cells both during hematopoiesis in the bone marrow as well as during microbe and DAMP exposure in the periphery. Thus, appropriate levels of circulating vitamin D should prevent the activation of self-reactive lymphocytes (step 1).

Improving the vitamin D status via vitamin D_3_ supplementation after MS diagnosis seems to be beneficial, since it results on the cellular level in the reduction of T_H_1 and T_H_17 blood counts [88] as well as in higher levels of T_reg_ cells [89]. This effect is also seen by the reduction in IL17 levels and an increase in the IL10 concentrations, which are the major cytokines produced by both cell types. Thus, the clinical result of improved 25(OH)D_3_ serum levels are reduced effector function of T_H_1 and T_H_17 cells, such as less destruction of the blood-brain barrier (step 2), less chronic inflammation and increased immunological tolerance, i.e., controlled effector functions of immune cell inactivating the CNS (step 3) and reduced hindering of oligodendrocyte precursor cell survival and differentiation for axon repair (step 4). Intervention studies performed in different countries with in total more than 1000 MS patients indicated a 50–70% decreased number of relapses, when the vitamin D status was above 50 nM [15]. A very recent systematic review of 15 RCTs summarized that although supplementation with vitamin D_3_ has a beneficial effect against new lesions, there is no significant evidence that it is effective for preventing relapses or the progression of MS [90]. However, all of these studies were shorter than the 5 years of the VITAL trial [29]. Moreover, there was no personalized daily vitamin D_3_ supplementation dose based on a vitamin D response index segregation. Therefore, there is a need for new vitamin D_3_ supplementation trials in MS where dosing is individualized and based on the responsiveness to the vitamin.

## 6. Conclusions and Perspectives

Vitamin D_3_ controls via its biologically active metabolite 1,25(OH)_2_D_3_ the VDR cistrome. The action of VDR results in epigenetic memory in respective cell types, such as T cells and monocytes. A smaller subset of these VDR binding sites represent enhancer regions that regulate the expression of target genes with key impact on immune functions like those of the *HLA* or *CXCL* gene cluster. In persons with a sufficient vitamin D status in relation to their vitamin D response index, VDR will be optimally activated. These individuals will benefit from a well-functioning immune system and therefore have a low risk in developing MS (Table 1). In contrast, in insufficiently supplemented low vitamin D responders immune cells function suboptimal and/or differentiate into MS promoting subtypes, such as T_H_1 and T_H_17 instead of T_H_2 and T_reg_ cells.

Some 25% of the general population are low vitamin D responders [45] (Section 2) and have an increased risk of developing MS. Therefore, future RCTs should consider the segregation into high, mid and low vitamin D responders. In addition, the guidelines for vitamin D supplementation should take into account that an individual may be a low vitamin D responder, i.e., higher daily doses should be recommended.

It is likely that low responsiveness to vitamin D is one of many approaches to characterize a subgroup of people that shows a susceptibility to multiple types of diseases including MS that is significantly higher than that of other subgroups. Thus, a low vitamin D index is a warning sign for the respective individual to better adapt his/her lifestyle to the given environmental conditions, such as (i) exposure to sunlight, (ii) sufficient physical activity outdoors and (iii) vitamin D_3_ supplementation in optimal amounts.

## Figures and Tables

**Table 1:** Previous clinical trials on vitamin D in MS (summarized in [90]) have not taken into the account the need for a dose adjustment according to the individual response index. Therefore, results were of mixed clinical efficacy. New vitamin D_3_ supplementation trials in MS should consider different dosing based on the patients response index to this allowing thus allowing a proper assessment of the biological and clinical effects.

Vitamin D Response Index	Daily Vitamin D_3_ Supplementation	Immune System Balance Restored	Clinical Effect
High	Standard (1000 IU)	+	Present
Mid	Standard (1000 IU)	+/−	Partial
Mid	Mid (2000 IU)	+	Present
Low	Standard (1000 IU)	−	Absent
Low	High (4000 IU)	+	Present

## Data Availability

Not applicable.
